# Standardized outcome measures for pregnancy and childbirth, an ICHOM proposal

**DOI:** 10.1186/s12913-018-3732-3

**Published:** 2018-12-11

**Authors:** Malini Anand Nijagal, Stephanie Wissig, Caleb Stowell, Elizabeth Olson, Isis Amer-Wahlin, Gouke Bonsel, Allyson Brooks, Matthew Coleman, Shamala Devi Karalasingam, James M N Duffy, Tracy Flanagan, Stefan Gebhardt, Meridith E Greene, Floris Groenendaal, J Ravichandran R Jeganathan, Tessa Kowaliw, Marije Lamain-de-Ruiter, Elliott Main, Michelle Owens, Rod Petersen, Irwin Reiss, Carol Sakala, Anna Maria Speciale, Rachel Thompson, Oluwakemi Okunade, Arie Franx

**Affiliations:** 1 University of California, Zuckerberg San Francisco General Hospital, San Francisco, CA USA; 2 International Consortium for Health Outcomes Measurement, Cambridge, MA USA; 3 Karolinska Institutet, Stockholm, Sweden; 4 Erasmus Medical Center, Rotterdam, Netherlands; 5 Hoag Memorial Hospital Presbyterian, Newport Beach, CA USA; 6 University Hospital Southampton, Hampshire, UK; 7 National Clinical Research Centre, Ministry of Health, Kuala Lumpur, Malaysia; 8 Balliol College, University of Oxford, Oxford, UK; 9 Nuffield Department of Primary Care Health Sciences, University of Oxford, Oxford, UK; 10 Kaiser Permanente, Richmond, CA USA; 11 Stellenbosch University and Tygerberg Hospital, Cape Town, South Africa; 12 Massachusetts General Hospital, Boston, MA USA; 13 University Medical Center Utrecht, Utrecht, Netherlands; 14 Sultanah Aminah Hospital, Johor Ministry of Health, Johor Bahru, Malaysia; 15 South Australian Maternity Reform Association (SAMRA) Inc, Adelaide, Australia; 16 California Maternal Quality Care Collaborative, Stanford, CA USA; 17 University of Mississippi Medical Center, Jackson, MS USA; 18 Women and Children’s Health Network, North Adelaide, South Australia; 19 National Partnership for Women & Families, Washington, D.C., USA; 20 American College of Nurse Midwives, Silver Spring, MD USA; 21 The Dartmouth Institute for Health Policy and Clinical Practice, Lebanon, NH USA; 22 Wilhelmina Children’s Hospital, University Medical Center Utrecht, Utrecht, 3508 AB The Netherlands; 23 University of Maryland School of Medicine, Baltimore, MD 21201 USA

**Keywords:** Health outcomes, Pregnancy, Obstetrics, Consensus, Delivery outcomes, Outcome measures, Perinatal health, DELPHI process, Patient-centred outcomes, Patient-reported

## Abstract

**Background:**

Value-based health care aims to optimize the balance of patient outcomes and health care costs. To improve value in perinatal care using this strategy, standard outcomes must first be defined. The objective of this work was to define a minimum, internationally appropriate set of outcome measures for evaluating and improving perinatal care with a focus on outcomes that matter to women and their families.

**Methods:**

An interdisciplinary and international Working Group was assembled. Existing literature and current measurement initiatives were reviewed. Serial guided discussions and validation surveys provided consumer input. A series of nine teleconferences, incorporating a modified Delphi process, were held to reach consensus on the proposed Standard Set.

**Results:**

The Working Group selected 24 outcome measures to evaluate care during pregnancy and up to 6 months postpartum. These include clinical outcomes such as maternal and neonatal mortality and morbidity, stillbirth, preterm birth, birth injury and patient-reported outcome measures (PROMs) that assess health-related quality of life (HRQoL), mental health, mother-infant bonding, confidence and success with breastfeeding, incontinence, and satisfaction with care and birth experience. To support analysis of these outcome measures, pertinent baseline characteristics and risk factor metrics were also defined.

**Conclusions:**

We propose a set of outcome measures for evaluating the care that women and infants receive during pregnancy and the postpartum period. While validation and refinement via pilot implementation projects are needed, we view this as an important initial step towards value-based improvements in care.

**Electronic supplementary material:**

The online version of this article (10.1186/s12913-018-3732-3) contains supplementary material, which is available to authorized users.

## Background

Maternity care is rife with unwarranted variation. Recommendations for optimal prenatal care and childbirth practices vary, even among advanced economies. Similarly, the use of common interventions such as induction of labor, continuous electronic fetal monitoring and cesarean section is variable [1–3]. There are also dramatic differences in the cost of maternity care: in 2015, the average standardized price to consumers of an uncomplicated birth in US dollars was $5312 in Australia, as compared to $10,808 in the United States [4]. Such variation presents an opportunity for health systems to learn from each other in their efforts to improve efficiency and effectiveness of clinical care. However, for this learning to occur, a standardized framework for evaluating pregnancy and postpartum care must be established. Value-based health care (VBHC) provides such a framework [5]. It defines value as the ratio of the outcomes of care divided by the cost of achieving those outcomes, with outcomes defined as the relevant end results of care from the perspective of the patient. By promoting the comparison of outcomes and costs of care using standardized metrics, VBHC enables providers and others delivering care to understand best practices for delivering high-value care to women and their infants [5].

A key challenge to applying the VBHC framework to pregnancy and childbirth has been the lack of standardized outcome measures in the field. Most commonly collected quality metrics in maternity care focus on health care processes such as rates of cesarean sections and prenatal care utilization. But, such measures do not directly capture the outcomes of pregnancy and childbirth foremost in most women’s minds – a healthy infant and healthy mother [6–8]. Furthermore, operational definitions for existing outcome measures vary considerably. For example, postpartum hemorrhage may be defined by the volume of blood loss [9, 10] or the need for the transfusion of blood products [11, 12]. Standardized, woman- and newborn-centered outcome measures, including both clinical outcomes and patient-reported outcomes (PROs), are needed to enable the use of VBHC to improve pregnancy and postpartum care.

The International Consortium for Health Outcomes Measurement (ICHOM) is a not-for-profit organization that aims to facilitate the adoption of value-based health care worldwide. As a first step in this process, it convenes international Working Groups of clinicians, researchers, and patients (“consumers”) to define standardized outcome measure sets for evaluating value in specific condition areas, with a focus on the outcomes that matter most to patients (www.ichom.org) [13]. The objective of the work presented here, initiated by ICHOM, was to recommend a minimum standard set of outcome measures and associated case-mix factors to be collected during the pregnancy and postpartum/newborn periods, to assist health systems with evaluating and improving the value of care they deliver.

## Methods

### Working group assembly and composition

ICHOM convened a Working Group composed of two consumer representatives and 19 international experts in various fields of perinatal and neonatal care, research and patient advocacy. Within the realm of feasibility, Working Group members were selected to provide balanced expertise across geographies and clinical specialties, as well as representation from obstetric registries and outcomes measurement initiatives (Table 1). The activities of the Working Group were coordinated by a Project Team consisting of a Working Group lead (Franx), a Project Lead (Wissig), a Research Fellow (Nijagal), and the ICHOM Vice President of Research & Development (Stowell).

**Table 1:** Working Group members by country and specialty, including organizations and data initiatives represented

Country	Specialty	Working Group member	Organization	Data initiatives
Australia	Consumer Representative	Tessa Kowaliw	South Australian Maternity Reform Association (SAMRA) Inc.	
	Obstetrics and Gynecology	Rod Petersen	Women and Children’s Health Network	
Italy	Midwifery	Anna Marie Speciale	American College of Nurse-Midwives	
Malaysia	Obstetrics and Gynecology	J Ravichandran R Jeganathan	Sultanah Aminah Hospital, Johor Ministry of Health, Malaysia	National Obstetrics Registry
		Shamala Devi Karalasingam	National Clinical Resarch Centre, Ministry of Health Malaysia	National Obstetrics Registry
Netherlands	Midwifery	Marije Lamain-de Ruiter	University Medical Center Utrecht	
	Neonatology	Floris Groenendaal	University Medical Center Utrecht	
		Irwin Reiss	Erasmus Medical Center	
	Obstetrics and Gynecology	Gouke Bonsel	Erasmus Medical Center	Mind2Care Foundation
		Arie Franx	University Medical Center Utrecht	Indicators Committee of the Dutch Society of Obstetrics and Gynecology (NVOG)
				Netherlands Perinatal Registry (PRN-foundation)
South Africa	Obstetrics and Gynecology	Stefan Gebhardt	Stellenbosch University and Tygerberg Hospital	
Sweden	Obstetrics and Gynecology	Isis Amer-Wahlin	Karolinska Institute	
United Kingdom	Obstetrics	Matthew Coleman	University Hospital Southampton	
		James Duffy	Balliol College, University of Oxford	Core Outcomes in Women’s Health (CROWN) initiative
United States	Consumer Representative	Meridith Greene	Massachusetts General Hospital	
	Health Policy	Carol Sakala	National Partnership for Women & Families	National Quality Forum’s (NQF): • MAP Medicaid Child Health Task Force • Perinatal and Reproductive Health Standing Committee
	Health Psychology	Rachel Thompson	The Dartmouth Institute for Health Policy and Clinical Practice	The Queensland Center for Mothers and Babies
	Maternal and Fetal Medicine	Elliott Main	CMQCC (California Maternal Quality Care Collaborative)	California Maternal Data Center (CMDC)
		Marlin Mills	Hoag Memorial Hospital	
		Michelle Owens	University of Mississippi Medical Center, ACOG	
	Obstetrics and Gynecology	Allyson Brooks	Women’s Health Institute at Hoag Memorial Hospital Presbyterian	
		Tracy Flanagan	Kaiser Permanente	
		Malini Nijagal	University of California San Francisco, Zuckerberg San Francisco General Hospital	

### Work process and decision-making

The measure set was developed using a modified Delphi method [14]. Between May 2015 and May 2016, the Working Group convened for nine teleconferences. Excluding the launch and final meetings, each teleconference had a pre-determined, specific goal such as establishing the scope of the measure set, defining the patient population, selecting outcomes and case-mix domains, identifying appropriate definitions and/or measures for each domain, and determining when each measure would be assessed during the pregnancy and postpartum course. Based on the goal, the Project Team reviewed relevant literature and current practices prior to the teleconference and presented this information, along with a specific proposal, during the teleconference for group discussion. Detailed minutes of these discussions were distributed following each teleconference to Working Group members, who then voted on each item of the Project Team’s proposal via an online survey. Items required a 70% agreement among survey respondents to be finalized into the measure set. Survey items with less than 70% majority were either excluded from the set or revised by the project team and re-presented for discussion and voting at the next teleconference.

### Selection of outcome domains, measures, and case-mix factors

Multiple information sources were sought to support the consideration of outcome domains to be included. In addition to reviewing outcomes included in regional perinatal health registries and quality indicator sets, a comprehensive literature review was performed using search terms focused on quality outcomes or indicators, birth experience and health-related quality of life (HRQoL). This resulted in a comprehensive list of both clinical and woman-centered outcomes. [Additional file 1]. A serial guided discussion among five pregnant and postpartum women was also conducted to identify additional outcomes that had not emerged from the literature search. Participants in this focus group were asked to reflect on their most significant experiences during the pregnancy, birth and postpartum periods as a mechanism to explore what participants’ felt were their most important goals of care. The group represented a variety of ages, parities, phases in the care cycle (prenatal vs. postpartum), clinical experiences (routine vs. complicated), and nationalities. We recognize that this did not provide a representative sample of pregnant and postpartum women globally; however, our aim was to gather further information to support decision-making and guide prioritization of outcome domains by the Working Group.

The comprehensive list of potential outcome domains was presented to the Working Group for discussion during a teleconference meeting. Working Group members were then asked to score each potential outcome on the GRADE scale via electronic survey [15]. Outcome domains thought to be “critical” (scored between 7 and 9) by at least 70% of the respondents were included in the set. Those scored as “low importance” (between 1 and 3) by at least 70% of respondents were excluded. The remaining domains were modified and re-presented for a second round of voting. Domains meeting neither the inclusion nor exclusion criterion after a second round of voting were discussed again by the Working Group and then presented for a final binary vote.

A similar protocol was followed to define appropriate measures for each domain, and to select the case-mix factors included in the set. Prior to teleconferences, the project team reviewed the literature to identify potential measures for each domain, and to compile a comprehensive list of demographic, social, and clinical factors associated with the selected outcomes. The final outcome measures and case-mix factors were then finalized through the process of Working Group discussion via teleconference, followed by voting via electronic survey.

### Determining timeline and process for measurement

To determine when and how each outcome measure and case-mix factor would be assessed during the pregnancy to postpartum continuum, the Project Team used the same process: current practices were researched, options discussed with the Working Group during teleconferences, and electronic surveys were administered for voting.

### Consumer validation surveys

To ensure robust consumer input in the development of the measure set, we solicited feedback from pregnant and postpartum women around the world via an anonymous online survey. Quorum Review IRB issued a written determination of exemption for the ICHOM Patient Advisory Group in Pregnancy and Childbirth. A link to the survey was distributed within Working Group members’ networks via social media, with no inclusion or exclusion criteria for participation. The survey presented, in lay terms, the outcome domains voted in for inclusion by the Working Group. Respondents were asked to score included domains according to their importance on the GRADE scale and were given an opportunity at the end of the survey to suggest any missing outcomes. Survey responses and suggestions were presented to the Working Group to inform their conclusion on the generalizability of the consumer advisory group discussion themes.

### Open review process

To also allow for input from healthcare professional stakeholders outside of the formal Working Group, a 4-week open review period was held prior to the last Working Group teleconference. The Project Team identified key stakeholders representing provider organizations, payers, consumer advocacies, and other individuals expressing interest in the measure set via the ICHOM website. Each was sent an overview of the set with links to the full detail Reference Guide and a feedback survey. The results of this survey were presented to the Working Group during the final teleconference call.

## Results

### Response rates

Response rates for the seven post-teleconference surveys present to Working Group members were 82, 82, 73, 73, 77, 77 and 73% respectively. Group size fluctuated due to the late addition of some members and occasional unavailability of others. All members received call minutes and were kept abreast of the Working Group’s progress. For post-teleconference surveys that involved two rounds of voting, the response rate for the second round is presented here.

### Scope

The measure set covers key outcomes of care for all women and their infants from the first prenatal visit through six months postpartum. The endpoint was selected as a pragmatic compromise: the Working Group recognized that while important outcomes may not emerge until later than 6 months after birth [16], the response rates for patient questionnaires decreases over time and, therefore, a much later endpoint may not be feasible [17, 18]. Pregnancies with pre- or postnatally diagnosed significant congenital anomalies are excluded from measurement.

### Patient focus group discussion

All five participants had one or more children; one was pregnant at the time of the discussion and four were postpartum.

Seven major themes emerged from the discussion.

1. The importance of having access to trusted information.
2. A desire to be involved in shared decision making.
3. A desire for immediate contact with their baby after delivery.
4. Mental health during the pre- and postnatal periods.
5. Anxiety about early pregnancy loss and the health of the unborn child in the first trimester.
6. A need for greater breastfeeding support.
7. Concerns about adapting to their new role as a mother.

These themes were presented to the working group during the second teleconference call.

### Outcome domains and measures

Outcome domains and definitions/measures included in the set are presented in Table 2, along with the percentage of responding Working Group members who agreed with the inclusion of the domain. Domains and measures for which there was significant discussion within the Working Group are discussed below.

**Table 2:** Outcome domains and definitions included in the Standard Set

Category and outcome domain	Outcome definition/measure	Data Source	Agreement^a^
Survival			
Maternal death	Death of a female from any cause related to or aggravated by pregnancy or its management (excluding accidental or incidental causes) during pregnancy and childbirth or within 42 days of pregnancy termination, irrespective of site or duration of the pregnancy ^b^	A	94%
Still birth	Pregnancy loss at or after 28 + 0 weeks gestation of a birth weight of greater or equal to 1000 g	A	87%
Neonatal death	Death of a live born neonate up to 28 days of life	A	100%
Severe Maternal Morbidity			
Maternal need for intensive care	Admission to an ICU or a unit that provides 24-h medical supervision and is able to provide mechanical ventilation or continuous vasoactive drug support at any point during pregnancy through 42 days postpartum for pregnancy or childbirth related complications.	A	100%
Maternal length of stay	Number of consecutive days in the hospital from delivery to discharge	A	100%
Late maternal complication	Admission or re-admission within the first 42 days postpartum for childbirth related complications ^c^	A	100%
Transfusion	Any transfusion of red blood cells within the first 42 days postpartum	A	100%
Neonatal Morbidity			
Spontaneous preterm birth	Live birth at < 37 +0 weeks gestation occurring after spontaneous labor or rupture of membranes	A	89%
Iatrogenic preterm birth	Cesarean or labor induction before < 37 weeks + 0 gestation excluding those occurring after spontaneous labor or rupture of membranes	A	89%
Oxygen dependence	Administration of O2 by any route for greater than 24 h at any point during the first 28 days of life	A	88%
Neonate length of stay	Number of consecutive days in hospital from birth through 28 days of life	A	88%
Birth injury	Subdural and cerebral hemorrhage, massive epicranial subaponeurotic hemorrhage, other injuries to skeleton due to birth trauma, injury to spine and spinal cord due to birth trauma, injury to brachial plexus due to birth trauma, other cranial and peripheral nerve injuries due to birth trauma in single live-born neonates	A	81%
Patient-reported Health Status			
Health related quality of life	Tracked via the PROMIS Global10	PR	81%
Incontinence	Tracked via either the ICIQ-SF or Wexner	PR	86%
Pain with intercourse	Tracked via PROMIS SFFAC102	PR	
Breastfeeding			
Success with breastfeeding	Please indicate how you are feeding your baby. My baby has received only breast milk in the past 7 days. This may include breast milk in a bottle/My baby has received a combination of breast milk, formula, or water in the past 7 days/My baby has received only formula, water, or other liquids but not breast milk in the past 7 days.	PR	83%
Confidence with breastfeeding	How confident do you feel about breastfeeding? Not at all confident/Not very confident/ Somewhat confident/Confident/Very confident.	PR	81%
	Option to track via the BSES-SF		72%
Role Transition			
Mother-infant attachment	Tracked via the MIBS	PR	72%
Confidence with role as a mother	How confident [will you feel when your baby is born/do you feel about looking after your baby]? Not at all confident/Not very confident/Somewhat confident/Confident/Very confident.	PR	94%
Mental Health			
Postpartum Depression	Assessed via the PHQ-2 with optional follow-up with the EPDS	PR	88%
Satisfaction with Care			
Satisfaction with the results of care	How satisfied are you with the results of your care during [your pregnancy/your labor and birth/the months after your baby was born]? Very unsatisfied/Unsatisfied/Neither satisfied nor dissatisfied/Satisfied/Very satisfied.	PR	81%
Healthcare Responsiveness			
Confidence as an active participant in healthcare decisions	Thinking about your care during [your pregnancy/your labor and birth/the months after your baby was born]… Were you given information about your choices for maternity care? Were you given enough information to help you decide about your care? Were you given information at the right time to help you decide about your care? No/To some extent/Yes	PR	94%
Confidence in healthcare providers	Do you have confidence and trust in the staff caring for you? No/To some extent/Yes.	PR	89%
Birth Experience			
Birth Experience	Assessed via the BSS_R	PR	100%

^a^Percentage agreement among survey respondents to include outcome domain in set

^b^This outcome should be tracked by all providers but will not be used for comparisons between providers or provider organizations

^c^Excludes initial hospitalization for childbirth

For data source: *A* administrative data, *PR* patient-reported data

#### Survival

Maternal mortality, stillbirth (fetal death), and neonatal death were considered key outcomes to include in the set, and the World Health Organization (WHO) definitions were selected as the international standard for each [19, 20]. However, low rates of maternal mortality within high-income countries may prohibit meaningful comparisons of this outcome between hospitals or health care provider organizations [11, 12, 21]. Therefore, we included maternal mortality in the measure set to encourage tracking and auditing of each case, but stipulate that rates should not be used for intra-national comparisons.

#### Morbidity

The working group unanimously voted to include the domain “severe maternal morbidity”; however, defining appropriate measures of this broad domain proved challenging. Most obstetric registries and regulatory bodies measure maternal morbidity by counting the occurrences of a comprehensive list of complications and adverse events, yet there is little consistency in which events are included [22, 23]. Furthermore, as with maternal mortality, rates of these events at individual hospitals or provider organizations are often too low to allow for meaningful comparisons.

Therefore, the Working Group selected four measures that represent the common endpoints of the leading causes of preventable maternal mortality worldwide, i.e. hypertensive disease, venous thromboembolism, sepsis, and obstetrical hemorrhage [24]. These included admission to an intensive care unit or transfer to another facility for intensive care, maternal length of stay, admission to the hospital during the postpartum period (i.e. readmission), and postpartum blood transfusions. These proxy measures aggregate across complications and adverse events to provide simple, standardized metrics for comparisons. The Working Group recognizes that the incidence of specific complications and adverse events must be tracked to properly interpret these proxy outcomes. In addition, although similar measures have been shown to correlate well with more traditional measures of maternal morbidity, the Working Group recommends testing and evaluation of these measures before broad adoption [25].

Similar rationale motivated the selection of measures to represent severe newborn morbidity: newborn length of stay (corrected for prematurity) and oxygen dependency for greater than 24 h. The Working Group felt that significant morbidity would be better measured in an international setting using oxygen dependency rather than neonatal intensive care unit (NICU) admission, as no universally accepted definitions for NICU levels exist and NICU use varies based on local circumstances and resources. This is even the case in a small country such as The Netherlands (www.perined.nl) where the presence or absence of intermediate care units leads to different criteria for admission to the NICU between tertiary hospitals. The outcomes of preterm birth and birth injury were also included in the measure set. Preterm birth, the leading cause of infant morbidity and mortality, is separated into spontaneous and iatrogenic (e.g. in case of severe maternal disease), as higher than expected rates of either may signify areas for improvement [26]. For birth injury, an inclusive definition was selected to include clavicular and brachial plexus injuries in addition to other more severe injuries, as these are not uncommon, may have significant long-term consequences for infants, and are distressing to families [27–29].

#### Domains representing patient-reported health and well-being

Overall health and wellbeing measures are most appropriately captured by self-report using Patient Reported Outcome Measures (PROMs). However, little work has been done on the use of PROMs in routine maternity care and none of the registries reviewed for this work include patient-reported measures [30]. To recommend measures for these important outcomes, we relied on PROMs that have been shown to successfully measure the outcome of interest in a general, non-maternity population (e.g. the Patient-Reported Outcomes Measurement Information System (PROMIS) Global to measure HRQoL, and the Patient Health Questionnaire-2 (PHQ-2) to measure postpartum depression) or that have proven useful in research studies (e.g. the Mother-Infant Bonding Scale (MIBS) to assess mother-infant attachment and the Breastfeeding Self-Efficacy Scale – Short Form (BSES-SF) to identify women struggling with breastfeeding). In some cases, individual questions were modified from maternity specific regional or national surveys, such as the National Perinatal Epidemiology Unit in the UK and the Queensland Centre for Mothers & Babies in Australia [31, 32]. Validated PROMs were selected based on their domain coverage, psychometric properties, validity, feasibility to implement and clinical interpretability, according to guidelines from the International Society for Quality of Life Research (ISOQOL) [33].

#### Birth experience

The quality of the birth experience was not an outcome originally voted for inclusion in the measure set by the Working Group. However, there was unanimous agreement to add satisfaction with the birth experience following analysis of the consumer validation survey responses. Notably, although 84% of validation survey respondents agreed that the set “captures the most important outcomes that matter or have mattered to you”, thematic analysis of free-text responses to the prompt “if not, what would you add” suggested a need to better understand the quality of the birth experience from the woman’s perspective. The Birth Satisfaction Scale - Revised (BSS-R), a validated 10-item questionnaire, was selected to capture this information [34]. (Details of the consumer validation survey are presented in Additional file 2.)

### Case-mix factors

A number of patient characteristics and risk factors are known to influence the outcomes presented above. To ensure fair comparisons across providers with diverse patient populations, the Working Group identified and defined key case-mix factors to include in the set. Factors selected for inclusion were considered to have a strong and independent effect on the outcomes included in the set, and to be practical for collection in an international setting. All case-mix factors and definitions are presented in Table 3, along with the percent of responding Working Group members who agreed upon their inclusion. The outcome of preterm birth also allows for stratification of other maternal and infant outcomes that may be impacted by gestational age at delivery.

**Table 3:** Case-mix variable domains and definitions included in the Standard Set

Category and case-mix factor domain	Case-mix factor definition	Data Source	Agreement^a^
Demographic Factors			
Age	Age at time of delivery	A	100%
Education level	Please indicate the highest level of schooling completed. None; Primary; Secondary; Tertiary (university or equivalent).	PR	94%
Race/ethnicity	Race/ethnicity as defined locally. Varies by country and should be determined by country (not for cross country comparison).	PR	88%
Social Support	SIMSS, How many people do you have near you that you can readily count on for help in time of difficulty such as to watch over children or pets, give rides to the hospital or store, or help when you are sick?	PR	75%
Parity	Have you given birth before? This includes both vaginal births and Cesarean sections (operations to remove your baby from your abdomen). Please do not count miscarriages or births that happened before 20 weeks (5 months) of pregnancy.	PR	100%
Obstetric and Medical History			
Obstetric history	If you have been pregnant before, have you experienced any of the following in previous pregnancies? Please mark all that apply. This is my first pregnancy. A baby born early, more than 3 weeks before his or her due date. Bleeding so much during pregnancy, birth, or after birth that you needed to be given blood. A Cesarean section (operation to remove your baby through your abdomen). Loss of a pregnancy after 20 weeks (5 months) of pregnancy.	PR	100%
Medical history	BEFORE you got pregnant, did a doctor, nurse, or other health worker tell you that you had any of the following health conditions? Tick all that apply: Diabetes; high blood pressure or hypertension; a mental health disorder such as depression, anxiety, bipolar disorder or schizophrenia.	PR	94%
Multiple gestations	Are you pregnant with: One baby, two babies (twins), three or more babies (triplets or higher).	PR	100%
BMI	What was your weight IMMEDIATELY before your pregnancy? (Weight in lbs. or kgs). What is your height? (Height in ft. or meters).	PR	94%
Substance use	Tobacco use, drug use, or alcohol use complicating pregnancy	PR	94%
Congenital anomaly	Diagnosis of a neonate with any of the following within 28 days of birth: Anencephaly, Spina bifida occulta, Meningo (myelo)cele, Hydrocephaly/holoprosencephaly without neural tube defect, Encephalocele, Neuromuscular abnormalities, Transposition of the great artieris, Tetralogy of Fallot, Hypoplastic left heart, Coarctation of the aorta, Complex cardiac malformation, Choanal atresia, Congenital malformation trachea, Lung hypoplasia, Hydro/Chylothorax, Congenital diaphragmatic hernia, Extrophia vesicase, Bilateral renal agenesis, Gastroschizis, Omphalocele, Trisomy 13, Trisomy 18, Trisomy 21, Congenital malignancy	A	94%
Treatment Variables			
Facility Type	Indicate where the birth took place (using local definitions for NICU levels): Birth at home or birth center, birth at a hospital with a level 1 or 2 NICU, birth at a hospital with a level 3 NICU.	A	94%
Route of delivery	Indicate the route of birth: spontaneous vaginal delivery, forceps or vacuum vaginal delivery, delivery by cesarean section.	PR	82%

^a^Percentage agreement among survey respondents to include case-mix factor

For data source: *A* administrative data, *PR* patient-reported data

### Timeline and process for measurement

**Fig. 1:**
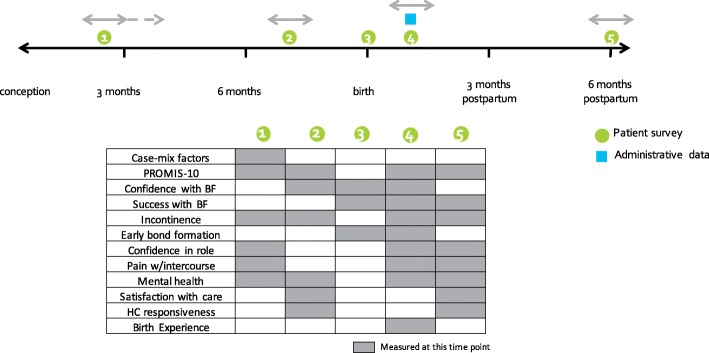
Timeline for ICHOM Pregnancy and Childbirth Standard Set data collection. The following timeline illustrates when Standard Set variables should be collected from patients, clinicians or administrative sources

The timeline for measurement was constructed based on clinical relevance and feasibility (Fig. 1). First, timeframes for measuring each outcome were identified based on clinical appropriateness. Next, recommended care schedules from several countries were analyzed to identify common time points at which women engage with maternity care. Tying patient-reported data collection to common clinic appointments allows collection to happen within the clinic and use of the data within clinical care. The 6-month postpartum data collection point is beyond the time frame of standard maternity care internationally and requires data to be collected from women via mail or electronic platforms.

Minimizing the length of patient surveys was a priority to reduce survey burden on women. Recognizing that not all women desire to breastfeed, the BSES-SF was made an optional measure to identify those who may benefit from additional support in the hospital or early postpartum period [35]. The Working Group also recommended a hierarchical question design when assessing outcomes affecting only a subset of women. For example, questions about the nature and frequency of urinary or fecal incontinence are burdensome for women without incontinence. Therefore, a single ICHOM-defined incontinence screening question is presented to all women with only those reporting symptoms going on to complete validated PROMs assessing symptom severity. Similarly, the Edinburgh Postnatal Depression Scale (EPDS) is included as an optional follow-up measure for those who screen positive on the shorter PHQ-2 [36–38]. Both measures have been validated for the pregnancy and the postnatal periods: the PHQ-2 is a practical and sensitive measure to detect perinatal depression, while the EPDS provides higher specificity [36]. Of note, while the Working Group advocated for postpartum depression screening by all maternity care providers, they emphasized that a response protocol must be in place to identify and treat individiuals who screen positive in a timely manner.

### Consumer validation surveys and open review feedback

A total of 105 consumer validation surveys and 17 complete responses to the open review feedback survey were received from across all continents except South America. Responses were generally positive. For the open review feedback, a median score of 4 (“agree”) on a 5-point scale from “strongly disagree” to “strongly agree” was obtained for statements about the scope of the measure set, the appropriateness of the included measures, and its ease of implementation. 94% of respondents reported that they would recommend implementation of the measure set to their colleagues. Specific survey comments were presented to the Working Group for discussion but resulted in no changes to the measure set.

## Discussion

The ICHOM Working Group on Pregnancy and Childbirth proposes a streamlined set of 24 outcome measures that are practical to measure, are internationally appropriate, and represent the goals of care that matter to women and their families. An associated set of case-mix factors is included to allow for outcome comparisons. We expect that measurement of these outcomes for every pregnancy, birth, woman and infant, when validated in diverse international settings, will facilitate communication between women and their care providers, incentivize and empower providers to improve care, and eventually, allow for benchmarking so that women and families, providers, and payers can make informed decisions about their health care spending and treatment options [39]. .Thus, we recommend this proposed measure set as an important step to achieving VBHC in pregnancy and postpartum care. A reference guide that includes the detailed measures, timeline for collection and patient-reported data questionnaires is publicly available through the ICHOM website to assist clinicians with starting measurement within their settings [40].

Of course, not all outcomes included in this set may be appropriate for making meaningful comparisons. In the case of rare outcomes, such as maternal mortality in developed countries, or outcomes that are determined largely by factors beyond care delivery processes, variation between providers may not be meaningful. Nevertheless, a comprehensive measure set that represents the most important outcomes from the perspective of women is critical for health systems to understand the overall goals of care and identify opportunities for improvement.

Measuring the outcomes in this set can immediately help healthcare providers both improve communication with patients and guide their quality improvement efforts. For example, urinary and/or fecal incontinence is experienced by up to 31% of women 6 months postpartum [41]; but despite a significant impact on health-related quality of life, many women do not report their symptoms [42, 43]. By giving women the opportunity to do so, patient-provider communication about this issue can improve and care options be explored. In addition, when measured on a large scale, providers may identify a need to change care processes that may contribute to this outcome.

As a result of our focus on outcomes that matter most to women, PROMs and patient-reported experience measures (PREMs) form a significant portion of the measure set. Traditionally, validated patient-reported measures have been used in the obstetrical research setting (e.g. to determine the prevalence of specific outcomes and evaluate their impact on HRQoL [42]) and within clinical practices on a limited basis (e.g. the EPDS) [38]. However, despite international interest in using patient-reported outcome measurement to drive clinical decisions and improve the care of individual patients, neither PROMs nor PREMs are included in any major perinatal registry or quality measure set that we reviewed [44]. We hope that our proposed measure set will facilitate the use of these measures more widely in maternity care.

Through this work, we also identify a set of case-mix factors to support the development of outcomes comparisons. The need for such a methodology in maternity care is well established [45]. Without appropriate risk adjustment, facilities may be reluctant to contribute data to benchmarking efforts or be transparent about their outcomes [46]. Some case-mix factors, such as obstetrical and medical history, may be most appropriately used to risk-adjust outcomes; others, such as facility type and delivery route, may be more appropriate to use in stratified outcome comparisons. Our identification of an evidence-based set of case-mix factors is an important step towards useful outcomes measurement and comparisons.

While our measure set focuses on outcomes of care, we do not suggest that process measurement should be abandoned. Evaluation of outcomes provides a framework for interpreting process data and identifying processes that can be improved. For example, multiple registries include “cesarean sections among low-risk mothers” as a quality metric in response to a concerning rise in the use of this procedure [8, 47, 48]. However, the optimal rate for this metric is unclear [49]. Assessments of overuse versus underuse of this procedure have been guided by the goal of preventing perinatal mortality and morbidity, but have not considered other important outcomes that may be impacted by the delivery route, such as time to recovery, difficulty with breastfeeding, and incontinence [50, 51]. By measuring a holistic set of outcomes in addition to cesarean rates, institutions can more comprehensively evaluate the impact of their cesarean rates on maternal and neonatal wellbeing.

### Strengths and limitations

Our work represents a unique contribution to health systems and providers seeking to improve perinatal care delivery. To our knowledge, this is the first internationally developed set of perinatal measures that: (a) focuses on outcomes that matter to women, rather than processes of care, (b) includes PROMs, and (c) includes a set of case-mix factors to facilitate outcome comparisons. By involving consumers in our work process and focusing on the goal of overall wellbeing of mothers and infants, we identified common pregnancy outcomes and experiences that may be overlooked by health care professionals, but have a major impact on physical and psychological wellbeing.

There were a number of limitations in this work. First, ICHOM aims to create measure sets that are appropriate across cultures, applicable in diverse health care settings, and practical to implement. However, for low-income, low-resource countries with high rates of mortality and high levels of morbidity, measurement of comprehensive perinatal outcomes may be less compelling and too burdensome at this time. Accordingly, although the Working Group represented a diverse range of middle- and high-income countries, representation from low-income countries was limited.

Second, it was challenging for the group to identify and agree on validated measures for each outcome domain. As discussed above, the Working Group unanimously agreed that severe maternal morbidity was an important outcome to include in the measures set. However, agreeing on the best measures to capture this outcome proved challenging. The “life-threatening condition” approach used by WHO was considered difficult to implement as it requires clinical report and may not be representative of severe morbidity in high-income countries [22]. In contrast, the approach used by the Center for Disease Control (CDC) [25] of using administrative data to track the incidence of 25 adverse maternal outcomes was considered too broad and cumbersome. In addition, the incidence of each of these adverse outcomes is typically quite low in advanced economies, limiting the use of this data for quality improvement [52]. As a compromise, the Working Group selected a handful of proxy measures (ICU admission, length of stay, pregnancy-related readmission, and blood transfusion) that are easily measured and have been shown to capture cases of significant adverse maternal outcomes [23, 53]. While these proxy measures may be considered processes rather than outcomes, each was considered an important outcome from the perspective of women as they each represent a delay in return to normal activity (prolonged facility stay), cause separation from their infant (ICU admission and postpartum readmission), or introduce new risk (blood transfusion).

Similar factors influenced the selection of other new or non-validated measures for inclusion in the set. The Working Group recognizes that these measures must be tested and validated over time, and ICHOM is committed to supporting this process. Implementors of measures in this set are encouraged to inform ICHOM of their work and share their experiences. A Steering Committee comprised of ICHOM Working Group members has been assembled to guide the continued maintenance and refinement of the set based on input from these early adopters. As measures are refined and implementation expands, ICHOM will work with implementors to validate measures as necessary.

Finally, the practicality of measurement and the burden of data collection in the clinical setting is always an important consideration. Although data abstracted from administrative records may have limited accuracy, capturing clinical data directly from providers is often prohibitively burdensome [54, 55]. Therefore, the measure set consists of a small number of administratively captured data points and relies heavily upon patient-reported data. This approach has proven successful in a variety of data collection efforts around the world [56–58], although capturing patient-reported outcomes remains a challenge, particularly in low- and middle- income countries. Along with helping a number of care delivery organizations with implementation of the measure set, ICHOM has partnered with PharmAccess Foundation to explore the possibility of using mobile phone technology to enable routine collection of patient-reported data in Kenya. While the number of electronic options for collecting such data continues to expand, distribution of paper surveys within the clinic remains a low-cost option [59].

## Conclusions

In conclusion, we expect that the introduction of this measure set will contribute significantly towards measuring and learning how to increase value in pregnancy and postpartum care. In time, providers and maternity care systems will be able to use such measures to identify effective, high-value practices across the pregnancy, childbirth and postpartum periods and to better target quality improvement efforts. Widespread measurement and reporting of this data will empower women as active participants in their care and enable consumers, providers, and payers to make better-informed decisions about health care options and spending helping to align incentives across these stakeholders.

## Additional files

Additional file 1: Overview of Systematic Literature Review. (PDF 542 kb)

Additional file 2: 2A. Patient Validation Survey: Patient characteristics of survey respondents; 2B. Patient Validation Survey: Results on score of importance of outcome domains. (PDF 32 kb)

## Abbreviations

BSES-SF: Breastfeeding self-efficacy scale – short form
BSS-R: Birth satisfaction scale – revised
EPDS: Edinburgh postnatal depression scale
HRQoL: Health-related quality of life
ICHOM: International consortium for health outcomes measurement
ICU: Intensive care unit
ISOQOL: International society for quality of life research
MIBS: Mother-infant bonding scale
NICU: Neonatal intensive care unit
PHQ-2: Patient health questionnaire-2
PREM: Patient-reported experience measure
PRO: Patient-reported outcome
PROM: Patient-reported outcome measure
PROMIS: Patient-reported outcomes measurement information system
VBHC: Value-based healthcare
WHO: World Health Organisation
