# Supplementary figures and images for: Standardized outcome measures for pregnancy and childbirth, an ICHOM proposal
Source: BMC Health Serv Res. 2018 Dec 11;18:953. doi: 10.1186/s12913-018-3732-3 (PMC6290550; doi:10.1186/s12913-018-3732-3)

## Additional file 1: Systematic Literature Review

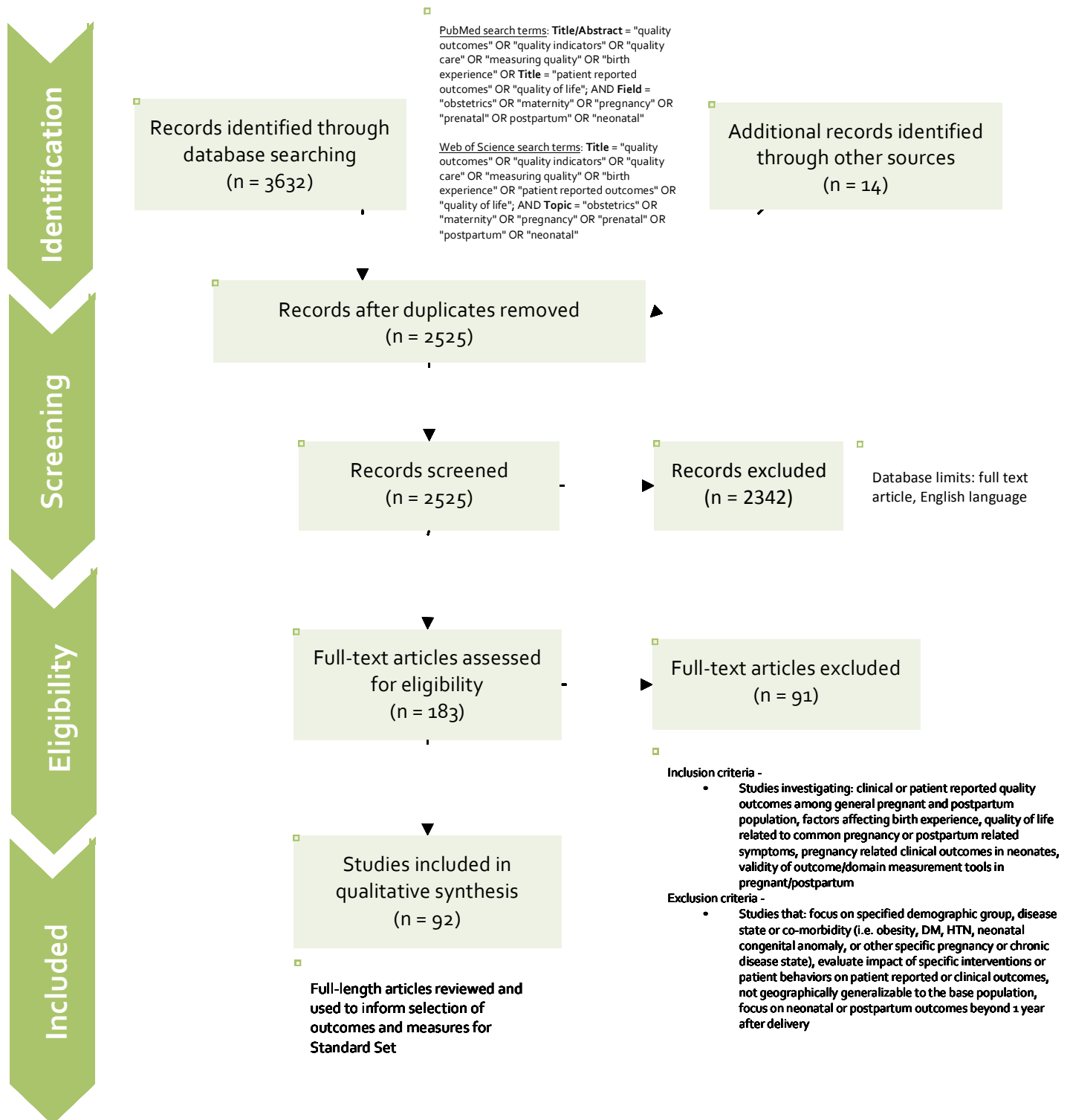

Supplement: Supplementary file 1 — Overview of Systematic Literature Review. (PDF 542 kb) [file 12913_2018_3732_MOESM1_ESM.pdf]
