# Supplementary material for: Standardized outcome measures for pregnancy and childbirth, an ICHOM proposal
Source: BMC Health Serv Res. 2018 Dec 11;18:953. doi: 10.1186/s12913-018-3732-3 (PMC6290550; doi:10.1186/s12913-018-3732-3)
Supplement: Supplementary file 2 — 2A. Patient Validation Survey: Patient characteristics of survey respondents; 2B. Patient Validation Survey: Results on score of importance of outcome domains. (PDF 32 kb) [file 12913_2018_3732_MOESM2_ESM.pdf]

Additional file 2A. Patient Validation Survey: Patient characteristics of survey respondents

Baseline characteristics	Survey respondents *N* = 105<br>%
Age, years	
20-25 years	13%
26-30 years	32%
31-35 years	38%
36-40 years	14%
>40 years	3%
Geography	
US	43.8%
Australia	49.5%
Europe (Germany, France, Norway)	5.7%
Stage of care cycle	
Currently preg	25.7%
Birth in last 6 mo	41.9%
Birth in > 6 mo	42.9%
1st pregnancy	25.7%
Satisfaction with care	
Satisfied	96 (91)
Unsatisfied	9 (9)
Experience of care	
Routine	62.9%
Complicated	37.1%

Additional file 2B. Patient validation survey: Results on score of importance of outcome domains.

Outcomes	% rating "very important" (score 7-9)	Mean score
Maternal death	83%	8.2
Still birth	84%	8.3
Neonatal death	85%	8.4
Major maternal complications	86%	8.1
Major neonatal complications	90%	8.3
Pre-term birth	74%	7.5
Birth injury	88%	8.2
Your general quality of life	62%	6.6
Confidence with breastfeeding	66%	6.8
Success with breastfeeding	64%	6.6
Postpartum depression	68%	6.8
Confidence in role	61%	6.5
Incontinence	56%	6.1
Pain with sex	52%	6.2
Mother-infant attachment	69%	6.7
Satisfaction with care	68%	6.9
Info, shared decisions, confidence in providers	71%	7.1

**Does this list capture all important outcome domains?**	
Agree	84%
Disagree	16%
**List of additional outcome domains:**	
Satisfaction with level of intervention	6
Informed choice	5
Respect	2
Emotional trauma/abuse	2
Continuity of care	2
Emotional support in post-partum period	2
Communication	2
Public reporting	1
